# The Frontal Assessment Battery (FAB) and its sub-scales: validation and updated normative data in an Italian population sample

**DOI:** 10.1007/s10072-021-05392-y

**Published:** 2021-06-29

**Authors:** Edoardo Nicolò Aiello, Antonella Esposito, Chiara Gramegna, Valentina Gazzaniga, Stefano Zago, Teresa Difonzo, Ildebrando Marco Appollonio, Nadia Bolognini

**Affiliations:** 1 School of Medicine and Surgery, University of Milano-Bicocca, Monza, Italy; 2 Clinical Neuroscience, University of Milano-Bicocca, Monza, Italy; 3 Department of Psychology, University of Milano-Bicocca, Milan, Italy; 4 Fondazione IRCCS Ca’ Granda Ospedale Maggiore Policlinico, University of Milan, Milan, Italy; 5 Milan Center for Neuroscience (NeuroMI), Milan, Italy; 6 Neurology Section, School of Medicine and Surgery, University of Milano-Bicocca, Monza, Italy; 7 Neuropsychological Laboratory, IRCCS Istituto Auxologico Italiano, Milan, Italy

**Keywords:** Frontal Assessment Battery, Frontal lobes, Executive functioning, Normative data, Dysexecutive symptoms, Neuropsychological assessment

## Abstract

**Background:**

Deficits of executive functioning (EF) are frequently found in neurological disorders. The Frontal Assessment Battery (FAB) is one of the most widespread and psychometrically robust EF screeners in clinical settings. However, in Italy, FAB norms date back to 15 years ago; moreover, its validity against “EF-loaded” global cognitive screeners (e.g., the Montreal Cognitive Assessment, MoCA) has yet to be tested. This study thus aimed at (a) providing updated normative data for the Italian FAB and (b) assessing its convergent validity with the MoCA.

**Methods:**

Four-hundred and seventy-five healthy Italian native speakers (306 females, 169 males; mean age: 61.08 ± 15.1; mean education: 11.67 ± 4.57) were administered by the MoCA and the FAB. FAB items were divided into three subscales: FAB-1 (linguistically mediated EF), FAB-2 (planning), and FAB-3 (inhibition). Regression-based norms were derived (equivalent scores) for all FAB measures.

**Results:**

Age and education were predictive of all FAB measures, whereas no gender differences were detected. The FAB and its sub-scales were related to MoCA measures—the strongest associations being found with MoCA total and MoCA-EF scores. FAB sub-scales were both internally related and associated with FAB total scores.

**Discussion:**

The FAB proved to have convergent validity with both global cognitive and EF measures in healthy individuals. The present study provides updated normative data for the FAB and its sub-scales in an Italian population sample, and thus supports an adaptive usage of this EF screener.

**Supplementary Information:**

The online version contains supplementary material available at 10.1007/s10072-021-05392-y.

## Introduction

Executive functioning (EF) comprises a multifaceted set of frontally mediated, noninstrumental cognitive processes that control instrumental domains and behavior [1]. Executive disorders are thus frequently found in a variety of neurological conditions of different etiologies that affect cortical/subcortical frontal structures [2].

Although second-level specific psychometric tests are to be preferred when assessing EF [3], screening instruments for executive deficits are often useful in clinical settings, such as providing with an optimal trade-off between informativity and both sensitivity and a rapid administration [4].

The Frontal Assessment Battery (FAB) [5] is an EF screener that requires 5–10’ to administer and consists of 6 tasks assessing different EF facets: (1) concept formation and abstract reasoning (similarities); (2) mental flexibility (phonemic verbal fluency); (3) motor programming (Luria motor sequences); (4) sensitivity to interference (conflicting instructions); (5) inhibitory control (go-no-go); (6) environmental autonomy (prehension behavior) [5, 6].

The FAB is one of the most widely used EF screeners worldwide; its psychometric properties, clinical usability, and neural correlates have been thoroughly investigated [7].

In Italy, the FAB has been adapted and normed, as well as validated in both healthy and clinical populations [6, 8]. However, current FAB Italian normative data date back to more than 15 years ago and sociodemographic changes require norms updating [9]. Moreover, to the best of the authors’ knowledge, the FAB has been only validated against “non-executive” screeners in Italy [6]—e.g., the Mini-Mental State Examination (MMSE) [10]—whereas its association with “EF-loaded” screening measures, e.g., the Montreal Cognitive Assessment (MoCA) [11], has yet to be explored. In addition, FAB normative studies do not provide with norms for its subtests, despite this being an increasingly widespread approach for cognitive screeners in Italy [12, 13], as it allows greater flexibility for clinicians when using these instruments.

Accordingly, the two aims of the present study are (1) providing updated normative data for both FAB total and sub-test scales in a large Italian representative population sample and (2) validating the FAB and its subscales against the MoCA.

## Subjects and methods

### Participants

The sample consisted of *N* = 475 healthy Italian native speakers from different provinces of Northern Italy (Table 1). Participants had no history of neurological, psychiatric, or severe general medical conditions (i.e. severe internal and metabolic morbidities or systemic/organ failures). Studies that data come from were approved by the Research Evaluation Committee of the Department of Psychology of University of Milano-Bicocca on behalf of the Ethical Committee of the same university. Participants provided informed consent to participation and signed a data treatment disclaimer research purposes.

**Table 1:** Sample stratification for age, education, and sex (M/F: M=males, F=females)

	Age (M/F)							
Education	35 ≤	36–45	46–55	56–65	66–75	76–85	86–95	> 95
5 ≤	0/0	0/1	0/0	0/3	1/18	11/33	4/5	1/0
6–11	1/1	6/5	9/26	20/15	5/10	6/21	4/2	0/0
12–16	6/7	4/8	20/42	31/33	7/5	5/9	1/6	0/0
17–21	3/5	1/7	6/13	17/22	0/1	1/2	0/2	0/0
> 21	0/0	0/2	0/2	0/0	0/0	0/0	0/0	0/0

Cells show male/female ratio for each co-occurrence

### Materials

Global cognition was assessed via the MoCA [12, 14], which encompasses subtests evaluating EF (MoCA-EF), attention (MoCA-A), language (MoCA-L), memory (MoCA-M), visuospatial functions (MoCA-VS), and orientation (MoCA-O). Supplementary Table 1 provides the protocol for the current FAB. FAB items (*N* = 6) were grouped into 3 subscales: FAB-1 comprising the first two items (similarities and phonemic verbal fluency, linguistically mediated EF); FAB-2, comprising the second two-item set (Luria motor sequences and conflicting instructions, planning); FAB-3, comprising the last two items (go-no-go and prehension behavior, inhibition). All participants were administered the MoCA first and then the FAB.

### Statistical analyses

According to previous normative studies [15, 16], the minimum sample size was set at *N* = 287 by means of a power analysis (*α* = 0.05; 1-*β* = 0.9; *f*^2^ = 0.05) for multiple linear regression (*df*_numerator_ = 3) analyses [17] via the R 3.6.3 package *pwr* [18].

Normality assumptions on both background and cognitive raw variables were checked by evaluating skewness and kurtosis values (judged as abnormal if ≥|1| and |3|, respectively) [19].

Associations of interest between quantitative variables were assessed by means of either Pearson’s or Spearman’s techniques. When judged as relevant, Bonferroni correction for multiple comparisons was applied.

According to the equivalent scores (ES) method [20, 21], raw scores (RSs) were adjusted for significant intervening background predictors (or their transforms) via regression-based equations. The cutoff was identified by computing outer and inner tolerance limits (oTL; iTL). Adjusted scores (ASs) were then standardized into a 5-level quasi-continuous scale: ES = 0 (ASs ≤ oTL; “abnormal”); ES = 4 (ASs > *Mdn*; “normal”); ES = 1, 2, and 3 (oTL < ASs ≤ *Mdn*; respectively, “borderline,” “low-end normal,” “normal”).

Analyses were performed via SPSS 27 [22] and R 3.6.3 [23]. Regression studies, as well as computations of both TLs and ES thresholds, were implemented according to guidelines and software solutions described in [24].

## Results

Background and cognitive scores are summarized in Table 2.

**Table 2:** Participants’ background and cognitive scores

*N*	Sex (F/M)	Age (years)	Education (years)	MoCA	FAB	FAB-1	FAB-2	FAB-3
475	306/169	61.08 ± 15.1 (21–96)	11.67 ± 4.57 (1–25)	24.5 ± 3.95 (8–30)	15.9 ± 2.17 (9–18)	5.21 ± 0.93 (1–6)	5.41 ± 1.08 (0–6)	5.27 ± 1.14 (1–6)

*F* female; *M* male; *MoCA* Montreal Cognitive Assessment; *FAB* Frontal Assessment Battery; FAB-1, comprises the first two items (similarities and phonemic verbal fluency); FAB-2, the second two-item set (Luria motor sequences and conflicting instructions); FAB-3, the last two items (go-no-go and prehension behavior)

The vast majority of FAB scales were related to MoCA measures (Table 3): the strongest associations were found with MoCA-EF and total scores. FAB subtest scores were all associated with each other (0.21 ≤ *r*_*s*_(475) ≤ 0.24; *p* < 0.001), as well as with FAB total scores (FAB-1: *r*_*s*_(475) = 0.65; FAB-2: *r*_*s*_(475) = 0.63; FAB-3: *r*_*s*_(475) = 0.7).

**Table 3:** Correlations between FAB and MoCA scores

		MoCA	MoCA-EF	MoCA-L	MoCA-A	MoCA-M	MoCA-VS	MoCA-O
FAB	*r*_*s*_	0.49	0.46	0.35	0.32	0.32	0.28	0.19
	*p*	< 0.001	< 0.001	< 0.001	< 0.001	< 0.001	< 0.001	< 0.001
FAB-1	*r*_*s*_	0.4	0.48	0.24	0.2	0.23	0.28	0.12
	*p*	< 0.001	< 0.001	< 0.001	< 0.001	< 0.001	< 0.001	n.s
FAB-2	*r*_*s*_	0.35	0.28	0.27	0.27	0.23	0.18	0.18
	*p*	< 0.001	< 0.001	< 0.001	< 0.001	< 0.001	< 0.001	< 0.001
FAB-3	*r*_*s*_	0.3	0.27	0.22	0.22	0.21	0.18	0.11
	*p*	< 0.001	< 0.001	< 0.001	< 0.001	< 0.001	< 0.001	n.s

*FAB* Frontal Assessment Battery (numbers following the acronym represent subtests); *MoCA* Montreal Cognitive Assessment; *EF* executive functioning; *L* language; *A* attention; *M* memory; *O* orientation; *VS* visuospatial. *α*_adjusted_ was set at 0.0017 (α/*k* = 0.05/28); *n.s.* not significant at *α*_adjusted_

Age was negatively related to FAB-1 (*r*_*s*_(475) =  − 0.3; *p* < 0.001), − 2 (*r*_*s*_(475) =  − 0.36; *p* < 0.001), − 3 (*r*_*s*_(475) =  − 0.3; *p* < 0.001) and total (*r*_*s*_(475) =  − 0.44; *p* < 0.001) scores, whereas a positive association with education was detected: FAB-1 (*r*_*s*_(475) = 0.35; *p* < 0.001), FAB-2 (*r*_*s*_(475) = 0.24; *p* < 0.001), FAB-3 (*r*_*s*_(475) = 0.31; *p* < 0.001), and total (*r*_*s*_(475) = 0.42; *p* < 0.001). No sex differences were found: FAB-1 (*t*(473) =  − 0.92; *p* = 0.357), FAB-2 (*t*(473) = 0.44; *p* = 0.66), FAB-3 (*t*(385.3) = 1.5; *p* = 0.25), and total (*t*(473) = 0.4; *p* = 0.689).

When simultaneously tested, age and education revealed to be predictive of FAB both total and subtest scores (age: − 0.17 ≤ *β* ≤  − 0.34; *p* ≤ 0.001; education: |0.15|≤ *β* ≤|0.33|; *p* ≤ 0.001). Cubic age and logarithmic education proved to be the most significant predictors of all FAB scales, with the exception of FAB-2 and FAB-3 that were best predicted by reciprocal education and quadratic age, respectively (Tables 4 and 5).

**Table 4:** Adjustment grid according to age and education for FAB total raw score

Education	Age												
	35	40	45	50	55	60	65	70	75	80	85	90	95
5	0.28	0.36	0.47	0.61	0.77	0.97	1.21	1.48	1.79	2.15	2.56	3.02	3.54
8	− 0.46	− 0.37	− 0.26	− 0.13	0.04	0.23	0.47	0.74	1.06	1.42	1.83	2.29	2.8
11	− 0.96	− 0.87	− 0.76	− 0.63	− 0.46	− 0.26	− 0.03	0.24	0.56	0.92	1.33	1.79	2.3
13	− 1.22	− 1.13	− 1.02	− 0.89	− 0.72	− 0.53	− 0.29	− 0.02	0.3	0.66	1.07	1.53	2.04
16	− 1.54	− 1.46	− 1.35	− 1.21	− 1.05	− 0.85	− 0.62	− 0.34	− 0.03	0.33	0.74	1.2	1.71
18	− 1.73	− 1.64	− 1.53	− 1.4	− 1.23	− 1.03	− 0.8	− 0.53	− 0.21	0.15	0.56	1.02	1.53
21	− 1.97	− 1.88	− 1.78	− 1.64	− 1.47	− 1.28	− 1.04	− 0.77	− 0.45	− 0.09	0.32	0.78	1.29

*FAB* Frontal Assessment Battery. Adjusted score** = **raw score + 0.000004*[(age^3)- 269,630.547368] -1.565729*[ln(education)-2.366383]. Significant decimals of adjustment factors are displayed. Adjustment factors have been extracted from the aforementioned formula and do not always reflect empirical co-occurrences

**Table 5:** Adjustment grids according to age and education for FAB-1, FAB-2, and FAB-3 raw scores

Subtest		Age												
FAB-1	Education	35	40	45	50	55	60	65	70	75	80	85	90	95
	5	0.23	0.25	0.28	0.32	0.36	0.41	0.46	0.53	0.61	0.7	0.8	0.92	1.05
	8	-0.05	-0.03	-	0.03	0.07	0.12	0.18	0.25	0.33	0.42	0.52	0.63	0.76
	11	-0.25	-0.22	-0.2	-0.16	-0.12	-0.07	-0.01	0.05	0.13	0.22	0.33	0.44	0.57
	13	-0.35	-0.33	-0.3	-0.27	-0.22	-0.17	-0.12	-0.05	0.03	0.12	0.22	0.34	0.47
	16	-0.47	-0.45	-0.43	-0.39	-0.35	-0.3	-0.24	-0.17	-0.09	-	0.1	0.21	0.34
	18	-0.54	-0.52	-0.5	-0.46	-0.42	-0.37	-0.31	-0.24	-0.17	-0.08	0.03	0.14	0.27
	21	-0.64	-0.62	-0.59	-0.56	-0.52	-0.47	-0.41	-0.34	-0.26	-0.17	-0.07	0.05	0.18
FAB-2	5	-0.21	-0.17	-0.12	-0.05	0.03	0.13	0.25	0.39	0.54	0.72	0.93	1.16	1.41
	8	-0.4	-0.36	-0.31	-0.24	-0.16	-0.06	0.06	0.2	0.35	0.53	0.74	0.97	1.23
	11	-0.49	-0.45	-0.39	-0.33	-0.24	-0.14	-0.03	0.11	0.27	0.45	0.65	0.88	1.14
	13	-0.53	-0.48	-0.43	-0.36	-0.28	-0.18	-0.06	0.07	0.23	0.41	0.62	0.85	1.1
	16	-0.56	-0.52	-0.47	-0.4	-0.31	-0.22	-0.1	0.04	0.2	0.38	0.58	0.81	1.07
	18	-0.58	-0.54	-0.48	-0.42	-0.33	-0.23	-0.12	0.02	0.18	0.36	0.56	0.79	1.05
	21	-0.6	-0.56	-0.5	-0.44	-0.35	-0.25	-0.14	-	0.16	0.34	0.54	0.77	1.03
FAB-3	5	0.2	0.24	0.29	0.33	0.39	0.45	0.51	0.58	0.66	0.74	0.82	0.91	1.01
	8	-0.1	-0.06	-0.02	0.03	0.09	0.15	0.21	0.28	0.36	0.44	0.52	0.61	0.71
	11	-0.3	-0.26	-0.22	-0.17	-0.12	-0.06	0.01	0.08	0.15	0.23	0.32	0.41	0.5
	13	-0.41	-0.37	-0.33	-0.28	-0.22	-0.16	-0.1	-0.03	0.04	0.12	0.21	0.3	0.39
	16	-0.54	-0.5	-0.46	-0.41	-0.36	-0.3	-0.23	-0.16	-0.09	-0.01	0.08	0.17	0.26
	18	-0.62	-0.58	-0.53	-0.49	-0.43	-0.37	-0.31	-0.24	-0.16	-0.08	-	0.1	0.19
	21	-0.72	-0.68	-0.63	-0.58	-0.53	-0.47	-0.41	-0.34	-0.26	-0.18	-0.1	-0.01	0.09

*FAB* Frontal Assessment Battery; the number following the acronym indicates the subscale in exam. FAB-1 adjusted score** = **raw score + 0.000001*[(age^3)-269,630.547368]-0.607345*[ln(education)-2.366383]. FAB-2 adjusted score** = **raw score + 0.000002*[(age^3)-269,630.547368] + 2.527494*[(1/education)-0.105356]. FAB-3 adjusted score** = **raw score + 0.000103*[(age^2)-3958.627368]-0.640471*[ln(education)-2.366383]. Significant decimals of adjustment factors are displayed. Adjustment factors have been extracted from the aforementioned formula and do not always reflect empirical co-occurrences

Selected correction factors and adjustment equations for FAB total and subtest RSs are displayed in Tables 4 and 5, respectively. TLs and ESs classifications for all FAB ASs are reported in Table 6.

**Table 6:** Equivalent Scores for the FAB-T, FAB-1, FAB-2, and FAB-3 adjusted scores

Equivalent scores							
	oTL	iTL	0	1	2	3	4
FAB	12.02	13.16	≤ 12.02	12.03–13.71	13.72–15.1	15.11–16.24	≥ 16.25
FAB-1	3.49	3.81	≤ 3.49	3.5–4.35	4.36–4.76	4.77–5.49	≥ 5.5
FAB-2	2.74	3.76	≤ 2.74	2.75–4.45	4.46–5.41	5.42–5.69	≥ 5.7
FAB-3	2.8	3.12	≤ 2.8	2.81–3.57	3.58–5.09	5.1–5.65	≥ 5.66

*FAB-T* Frontal Assessment Battery (numbers following the acronym represent subtests); *oTL* outer tolerance limit; *iTL* inner tolerance limit

## Discussion

The present work provides Italian practitioners with updated normative data for the FAB and its subtests. These norms cover a wider age and education range and are drawn from a larger sample size (*N* = 475) than those of previous normative studies (*N* = 236 [8] and *N* = 364 [6]). Moreover, norms for FAB subscales are provided—this representing a previously unreported feature that supports an adaptive usage of the screener.

This study overall replicates previous findings with respect to the range of FAB scores’ predictors in the Italian population: the performance increases with higher educational attainment and decreases with aging, while no sex differences are detected[6, 8]. It has nonetheless to be noted that the cutoff reported here (12.03), despite being similar to that derived by Iavarone et al. [8] (11.54), is more conservative than Appollonio et al.’s [6] (13.5). This aspect might reflect sociodemographic changes that have occurred in the last two decades in northern Italian population.

To the best of the authors’ knowledge, this contribution is the first showing convergent validity between the FAB (and its sub-scales) and an “EF-loaded” cognitive screener—i.e., the MoCA—in Italian healthy individuals. The present results also support the notion of the MoCA being a screening instrument sensitive to EF deficits, when compared to other screeners, such as the MMSE [25].

## Supplementary Information

Below is the link to the electronic supplementary material.

### Supplementary Information

Below is the link to the electronic supplementary material.

ESM1 (PDF 433 kb)

## Data Availability

Data collected and analyzed during the present study are available on the Open Science Framework (OSF) repository (https://osf.io/4xypv/).
