# Correction to: The Frontal Assessment Battery (FAB) and its sub‑scales: validation and updated normative data in an Italian population sample

**DOI:** 10.1007/s10072-022-06087-8

**Published:** 2022-04-22

**Authors:** Edoardo Nicolò Aiello, Antonella Esposito, Chiara Gramegna, Valentina Gazzaniga, Stefano Zago, Teresa Difonzo, Ildebrando Marco Appollonio, Nadia Bolognini

**Affiliations:** 1 School of Medicine and Surgery, University of Milano-Bicocca, Monza, Italy; 2 Clinical Neuroscience, University of Milano-Bicocca, Monza, Italy; 3 Department of Psychology, University of Milano-Bicocca, Milan, Italy; 4 Fondazione IRCCS Ca’ Granda Ospedale Maggiore Policlinico, University of Milan, Milan, Italy; 5 Milan Center for Neuroscience (NeuroMI), Milan, Italy; 6 Neurology Section, School of Medicine and Surgery, University of Milano-Bicocca, Monza, Italy; 7 Neuropsychological Laboratory, IRCCS Istituto Auxologico Italiano, Milan, Italy

**Correction to: Neurological Sciences (2022) 43:979–984**

**https://doi.org/10.1007/s10072-021-05392-y**

The original article contains an error. The affiliation 5 “Milan Center for Neuroscience (NeuroMI), Milan, Italy” should be remove from the author Nadia Bolognini’s address.

