# Supplementary material for: The Frontal Assessment Battery (FAB) and its sub-scales: validation and updated normative data in an Italian population sample
Source: Neurol Sci. 2021 Jun 29;43(2):979–84. doi: 10.1007/s10072-021-05392-y (PMC8789707; doi:10.1007/s10072-021-05392-y)
Supplement: Supplementary file 1 — (PDF 433 kb) [file 10072_2021_5392_MOESM1_ESM.pdf]

Frontal Assessment Battery (FAB) [1,2,3]

Aiello, E.N., Esposito, A., Gramegna, C., Gazzaniga, V., Zago, S., Difonzo, T., Appollonio, I., Bolognini, N. (2021). The Frontal Assessment Battery (FAB) and its sub-scales: validation and updated normative data in an Italian population sample. *Neurological Sciences*, 1-6. https://doi.org/10.1007/s10072-021-05392-y

1.a. “In che cosa sono simili una banana e un’arancia?” Risposta: ________ (“frutta”=1 pt.)
Note: in caso di fallimento totale “non sono simili” o di fallimento parziale “entrambe hanno la buccia” aiutare il S.: “la banana e l’arancia sono entrambe […]” ma assegnare 0 a questa risposta; non aiutare per i successivi due *item* (1.b e 1.c).
1.b. “In che cosa sono simili un tavolo e una sedia?” Risposta: ________ (“mobili”=1 pt.)
1.c. “In che cosa sono simili un tulipano, una rosa e una margherita?” Risposta: ________ (“fiori”=1 pt.) [__/3]

2. “Dica il maggior numero possibile di parole che cominciano con la lettera “S”, qualsiasi parola eccetto cognomi o nomi propri.” ***Note*:** la prova dura 60”; se il Soggetto (S.) non dà nessuna risposta nei primi 5”, dire: “Per esempio, “serpente” ”. Se il S. si ferma per oltre 10”, stimolarlo dicendo: “Qualsiasi parola che cominci con la lettera “S” ”. Ripetizioni o variazioni (ad es. scarpa, scarpone), cognomi o nomi propri non sono considerate risposte corrette.
Produzione:____
____
____
Attribuzione del punteggio: **3**=più di 9 parole; **2**=6-9 parole; **1**=3-5 parole; **0**=meno di 3 parole [__/3]

3. “Guardi con attenzione quello che faccio.” l’Esaminatore (E.) seduto di fronte al S. effettua 3 volte, da solo, con la mano sinistra la serie di Luria ***pugno-taglio-piatto***. *“*Ora faccia lo stesso anche lei prima con me, poi da solo*”*. l’E. effettua 3 volte la stessa serie con il S., poi gli dice: *“*continui da solo*”*.
Attribuzione del punteggio: **3**=il S. effettua da solo, correttamente, 6 serie consecutive; **2**=il S. effettua da solo, correttamente, almeno 3 serie consecutive; **1**=il S. sbaglia da solo, ma effettua correttamente almeno 3 serie consecutive con l’E.; **0**=il S. non riesce a effettuare 3 serie consecutive neppure con l’E. [__/3]

4. *“*Batta due volte quando io batto una volta.” ***Note*:** per essere sicuri che il S. abbia capito le istruzioni, si effettua una serie di 3 prove: “1-1-1”; Risposta: __-__-__
“Batta una volta quando io batto due volte.” ***Note*:** per essere sicuri che il S. abbia capito le istruzioni, si effettua una serie di 3 prove: “2-2-2”; Risposta: __-__-__
L’E. effettua la seguente serie: 1-1-2-1-2-2-2-1-1-2; Risposta: __-__-__-__-__-__-__-__-__-__
Attribuzione del punteggio: **3**=0 errori; **2**=1-2 errori; **1**=3 errori; **0**=più di 3 errori. [__/3]

5. *“*Batta una volta quando io batto una volta.” ***Note*:** per essere sicuri che il S. abbia capito le istruzioni, si effettua una serie di 3 prove: “1-1-1”; Risposta: __-__-__
“Non batta quando io batto due volte.” ***Note*:** per essere sicuri che il S. abbia capito le istruzioni, si effettua una serie di 3 prove: “2-2-2”; Risposta: __-__-__
L’E. effettua la seguente serie: 1-1-2-1-2-2-2-1-1-2; Risposta: __-__-__-__-__-__-__-__-__-__
Attribuzione del punteggio: **3**=0 errori; **2**=1-2 errori; **1**=3 errori; **0**=più di 3 errori. [__/3]

6. l’E. è seduto di fronte al S. Mettere le mani del S. con le palme in alto, appoggiate sulle ginocchia. Senza dire nulla e senza guardare il S., l’E. porta le sue mani vicino a quelle del S. e ne tocca le palme, contemporaneamente da ambo i lati, osservando se il S. spontaneamente le afferra. Se il S. le afferra, l’E. prova di nuovo dopo avergli detto: “Non prenda le mie mani”.
Attribuzione del punteggio: **3**=il S. non afferra le mani dell’E.; **2**=il S. esita e chiede cosa deve fare; **1**=il S. afferra le mani senza esitazione; **0**=il S. afferra le mani dell’E. anche dopo che gli è stato chiesto di non farlo. [__/3]

**FAB-1 (*item* 1 e 2): PG=__/6**	**PC=___**	**PE=___**
**FAB-2 (*item* 3 e 4): PG=__/6**	**PC=___**	**PE=___**
**FAB-3 (*item* 5 e 6): PG=__/6**	**PC=___**	**PE=___**
**FAB-tot.: PG=__/18**	**PC=___**	**PE=___**

[1] Dubois, B., Slachevsky, A., Litvan, I., Pillon, B. (2000). The FAB: a frontal assessment battery at bedside. *Neurology*, *55*, 1621-1626.
[2] Appollonio, I., Leone, M., Isella, V., Piamarta, F., Consoli, T., Villa, M.L., Forapani, E., Russo, A., Nichelli, P. (2005). The Frontal Assessment Battery (FAB): normative values in an Italian population sample. *Neurological Sciences*, *26*, 108-116.
[3] Barletta-Ghidolfi, C., Gasparini, F., Ghidoni, E. (2011). Kit del Neuropsicologo Italiano. Società Italiana di Neuropsicologia.
